# Pea Fiber and Wheat Bran Fiber Show Distinct Metabolic Profiles in Rats as Investigated by a ^1^H NMR-Based Metabolomic Approach

**DOI:** 10.1371/journal.pone.0115561

**Published:** 2014-12-26

**Authors:** Guangmang Liu, Liang Xiao, Tingting Fang, Yimin Cai, Gang Jia, Hua Zhao, Jing Wang, Xiaoling Chen, Caimei Wu

**Affiliations:** 1 Institute of Animal Nutrition, Sichuan Agricultural University, Chengdu, 611130, Sichuan, China; 2 Key Laboratory for Animal Disease-Resistance Nutrition of China Ministry of Education, Chengdu, 611130, Sichuan, China; 3 Japan International Research Center for Agricultural Sciences, 1-1 sukuba, Ohwashi, TIbaragi, 305-8686, Japan; 4 Maize Research Institute, Sichuan Agricultural University, Chengdu, 611130, Sichuan, China; University of East Anglia, United Kingdom

## Abstract

This study aimed to examine the effect of pea fiber (PF) and wheat bran fiber (WF) supplementation in rat metabolism. Rats were assigned randomly to one of three dietary groups and were given a basal diet containing 15% PF, 15% WF, or no supplemental fiber. Urine and plasma samples were analyzed by NMR-based metabolomics. PF significantly increased the plasma levels of 3-hydroxybutyrate, and *myo*-inositol as well as the urine levels of alanine, hydroxyphenylacetate, phenylacetyglycine, and α-ketoglutarate. However, PF significantly decreased the plasma levels of isoleucine, leucine, lactate, and pyruvate as well as the urine levels of allantoin, bile acids, and trigonelline. WF significantly increased the plasma levels of acetone, isobutyrate, lactate, *myo*-inositol, and lipids as well as the urine levels of alanine, lactate, dimethylglycine, *N*-methylniconamide, and α-ketoglutarate. However, WF significantly decreased the plasma levels of amino acids, and glucose as well as the urine levels of acetate, allantoin, citrate, creatine, hippurate, hydroxyphenylacetate, and trigonelline. Results suggest that PF and WF exposure can promote antioxidant activity and can exhibit common systemic metabolic changes, including lipid metabolism, energy metabolism, glycogenolysis and glycolysis metabolism, protein biosynthesis, and gut microbiota metabolism. PF can also decrease bile acid metabolism. These findings indicate that different fiber diet may cause differences in the biofluid profile in rats.

## Introduction

Dietary fiber (DF) has attracted considerable interest recently because many studies have uncovered its disease preventive and health-promoting functionalities, including blood cholesterol and/or glucose attenuation, laxative effect, and colon cancer, heart disease, and obesity risk reduction [1]. DF mainly consists of nonstarch polysaccharides (NSP) and lignin that pass into the colon where they are fermented by resident microbial bacteria communities [2], [3].The extent of fermentation depends on many factors such as solubility, structure, and degree of lignification of the fiber. Food with high soluble fiber content and low degree of lignification is prone to bacterial degradation and therefore has great influence on bacterial metabolism and production of short-chain fatty acids (SCFAs), such as acetate, propionate, and butyrate [4]. Compared with soluble fiber, insoluble fiber (i.e., cellulose and most pentosans) is fermented more slowly and thus increases the fecal bulk more. SCFAs function as an energy source and decrease the colonic pH, thereby promoting the growth of beneficial bacteria, such as *Bifidobacteria* and *Lactobacilli* [5]. These bacteria can interact with the host immune system [6], produce certain vitamins in the lumen [7], and promote gut architecture and function development [8]. Moreover, propionate has been shown to inhibit the synthesis of liver cholesterol [9]. Fibers can directly interfere with lipid absorption by changing the luminal solubility and the digestive processes.

Pea fiber (PF) and wheat bran fiber (WF) are increasingly incorporated into human food and animal diets as DF ingredients. Previous studies have shown that wheat bran, a by-product generated in large amounts during wheat processing, is a concentrated source of insoluble fiber, in which 46% is NSP. The main NSPs present are arabinoxylan, cellulose, and β-glucan (70%, 24%, and 6% of the NSP of the bran, respectively) [10].Wheat bran has shown antioxidant activities in vitro [11]. Some studies indicate that WF decreases fecal bile acid concentration and blood glucose and cholesterol levels in type 2 diabetic patients [12], [13]. PF may be an interesting DF source because PF is white, has good palatability, has high insoluble fiber, and has granulated powder that is easily baked into bread and meat [4]. The postprandial blood glucose response is markedly reduced by PF [14]. Pea has been shown to lower serum cholesterol levels [15]. Moreover, PF does not significantly alter the excretion of total bile acids, but decreases the concentration of fecal total bile acids [4]. The exact mechanisms by which PF and WF contribute to various health conditions are still not fully understood. Therefore, the health effects of PF and WF consumption and knowledge of these mechanisms need to be elucidated.

Recent metabolomics studies reveal the effects of exposure to whole grain wheat flours (compared with refined wheat flours) and to sweet potato fiber in the endogenous metabolism of rats [16], [17]. Rye fiber supplementation can alter the urine and plasma metabolome in pigs [18]. Moreover, the difference of the plasma metabolic profiles between low fiber and high fiber is shown in humans [19]. Thus, metabolomics can be considered an emerging and promising science with a level of information that spans the traditional approach for elucidating the biochemical response to diet and unrecognized mechanisms. However, no studies are available on the response of animal or human biological systems to PF supplementation, and few studies have focused on the response of animal or human biological systems to WF supplementation.

The rat model used has been shown to correlate with human studies [20]. These profiles provide evidence on the relationship between metabolites and nutritional biochemical mechanisms of PF and WF supplementation and establish the baseline data for future metabolomic studies. This approach is potentially useful to investigate PF and WF metabolism and verifies the association between PF and WF administration and health or disease risk. This study aims to investigate the effect of PF and WF supplementation in the urine and plasma composition of rats by using explorative metabolomic analyses through ^1^H NMR spectroscopy and chemometrics.

## Materials and Methods

The experimental protocol used in this study was approved by the Animal Care and Use Committee of Animal Nutrition Institute, Sichuan Agricultural University, and was carried out according to the National Research Council's Guide for the Care and Use of Laboratory Animals.

### Dietary fibers

PF and WF were purified from pea and wheat, respectively. PF was provided by Shandong Jianyuan Foods Co., Ltd. Shandong, China, whereas WF was from Sichuan Foods Co., Ltd. Sichuan, China. Table 1 lists the crude protein, crude fiber, neutral-detergent fiber, acid-detergent fiber, cellulose, hemicelluloses, lignin, soluble fiber, insoluble fiber, and total fiber contents of the fiber sources.

**Table 1:** Contents of the fiber sources.

Items^a^	Pea fiber	Wheat bran fiber
Crude protein (%)	10.4	17.1
Crude fiber (%)	23.6	10.1
Neutral-detergent fiber (%)	68.1	67.2
Acid-detergent fiber (%)	28.6	11.2
Cellulose (%)	26.9	8.7
Hemicellulose (%)	39.4	56.0
Lignin (%)	1.4	2.1
Soluble fiber (%)	3.9	6.2
Insoluble fiber (%)	59.3	46.3
Total fiber (%)	63.2	52.5

a These parameters were assayed in our laboratory.

### Animal experiment and sample collection

A total of 33 eight-week-old female Sprague–Dawley rats weighing 174 g to 202 g were housed in individual metabolic cages. The animals were allowed to acclimatize for a week. After this period, the rats were assigned randomly to three purified dietary groups, with 11 rats in each group, for 30 d. The first and second groups were fed a basal diet containing 15% PF and 15% WF, respectively. The third group was fed a basal diet without supplemental fiber source. The diets (Table 2) were formulated to meet the recommended nutrients of AIN 39 for laboratory rodents [21].To ensure similar gross energy levels in all diets, corn starch and soybean oil were decreased in the fiber-source diets. The bodyweight of each rat was determined once a week. The daily feed intake of the rats was also recorded. Urine samples were collected from each rat into ice-cooled vessels, containing 30 µL of sodium azide solution (1.0% w/v) from day 28 to day 29 of the treatment period (24 h). Blood samples were also collected (0900 a.m.) from the eye and placed into Eppendorf tubes with the addition of sodium heparin after anesthesia with ether at the end of the 30 d treatment period. Blood samples were centrifuged at 3500* g* for 10 min at 4°C to obtain plasma. All urine and plasma samples were stored at −80°C prior to preparation for NMR acquisition. The dosage selected for this experiment was based on previous study [22]. The rats were given ad libitum access to food and drinking water. The experimental conditions throughout the study were maintained at 22°C to 25°C with relative humidity within 50% to 70% in a 12 h light/12 h dark cycle. Clinical observations were performed during the experimental period.

**Table 2:** Composition and nutrient levels of the basal diet (as-fed basis).

Items			Fiber sources
	Control	Pea fiber	Wheat bran fiber
Ingredients (%)			
Cornstarch	44.74	31.92	31.92
Casein	20	20	20
Dextrinized cornstarch	13.2	13.2	13.2
Sucrose	10	10	10
Soybean oil	7	4.82	4.82
Pea fiber	0	15	0
Wheat bran fiber	0	0	15
L-Cystine	0.3	0.3	0.3
Mineral mix	1	1	1
Vitamine mix	3.5	3.5	3.5
Choline Chloride	0.246	0.246	0.246
Antioxidants	0.014	0.014	0.014
Total	100	100	100
Analysed chemical composition			
Gross energy (Kcal/kg)^a^	3766	3766	3766
Crude protein (%)^a^	16.6	16.6	16.6
Crude fiber (%)	0.52	5.59	2.50
Neutral-detergent fiber (%)	1.46	17.54	14.01
Acid-detergent fiber (%)	0.95	3.34	2.61
Cellulose (%)	0.55	1.54	0.88
Hemicellulose (%)	0.50	14.20	11.40
Lignin (%)	0.32	1.41	1.24
Soluble fiber (%)	3.7	1.8	5.3
Insoluble fiber (%)	2.7	7.9	3.6
Total fiber (%)	6.4	9.7	8.9

a calculated value.

### Clinical chemistry measurements

Clinical chemistry analyses were performed with an automatic biochemical analyzer (AUTOLAB PM 4000; AMS Corporation, Rome, Italy), which included total protein (TP), albumin (ALB), triglycerides (TG), total cholesterol, glucose, blood urea nitrogen (BUN), creatinine (CRE), aspirate aminotransferase (AST), and alanine aminotransferase (ALT).

### Sample preparation and NMR spectroscopy

Approximately 550 µL of aliquot from each urine sample was mixed with 55 µL of phosphate buffer (1.5 M NaH_2_PO_4_/K_2_HPO_4_, pH 7.4, 100% v/v D_2_O) with 5.0 mM 2,2-dimethyl-2-silapentane-5-sulfonate-d_6_ (DSS) as chemical shift reference (δ0.00 ppm) and 0.1% NaN_3_ as bacterial growth inhibitor. After centrifugation at 14000 g for 10 min, the supernatant was transferred into 5 mm NMR tubes for ^1^H NMR analysis. The plasma samples were prepared by mixing 200 µL of plasma with 400 µL of saline solution containing 75% D_2_O for field-frequency lock. After vortexating and centrifugation at 14000 g for 10 min at 4°C, 550 µL of samples was transferred into 5 mm NMR tubes.

The proton NMR spectra of the urine and plasma samples were acquired at 300 K on a Bruker Avance II 600 MHz spectrometer (Bruker Biospin, Rheinstetten, Germany) operating at a ^1^H frequency of 600.13 MHz with broadband-observe probe. For urine samples, a standard water-suppressed one-dimensional NMR spectrum was recorded by using the first increment of the gradient-selected NOESY pulse sequence (NOESYGPPR1D, recycle delay−90°−*t*_1_−90°−*t*_m_−90°−acquire data) with a recycle delay of 2 s, *t*_1_ of 3 µs, mixing time (*t*_m_) of 100 ms, and 90° pulse length of 13.70 µs. A total of 128 transients were collected into 49178 data points, employing a spectral width of 9590 Hz and an acquisition time of 2.56 s. A water-presaturated Carr–Purcell–Meiboom–Gill pulse sequence (recycle delay−90°−(*τ*−180°−*τ*)_n_−acquisition) was used to attenuate NMR signals from macromolecules. A spin–spin relaxation delay (2n*τ*) of 76.8 ms and a spin–echo delay *τ* of 400 µs were employed. The 90° pulse was set to 13.7 µs, and 32 transients were collected into 49178 data points for each spectrum with a spectral width of 15 ppm. Other acquisition parameters were the same as described above. All acquired free induction decays were multiplied by an exponential window function with a 1 Hz line-broadening factor before Fourier transformation. Metabolite assignments were generally made by considering the chemical shifts, coupling constants, and relative intensities as in previous studies. Additional assignment of the peaks to specific metabolites was on the basis of ^1^H–^1^H correlation spectroscopy and ^1^H–^1^H total correlation spectroscopy (data not shown).

### NMR spectroscopic processes and analysis

The NMR spectra were manually corrected for possible phase and baseline distortions. The plasma spectral region δ0.5–9.0 and the urinary spectral region δ0.5–9.5 were integrated into regions with an equal width of 0.002 and 0.01 ppm, respectively, by using Mestrenova 8.1.2 software (Mestrelab Research S.L., Spain). Plasma and urine chemical shifts were referenced to the peak of the methyl proton of L-lactate at δ1.33 and the peak of DSS at δ0.00, respectively. The ethanol signals originating from the process of blood collection were carefully excluded together with the regions containing urea and H_2_O signals to obtain the endogenous metabolite changes induced by PF and WF exposure. In the plasma spectra, the discarded regions include δ4.19–5.23 and δ5.40–6.04 for H_2_O and urea and δ1.16–1.19 and δ3.60–3.62 for ethanol. In the urine spectra, the discarded regions include δ4.50–5.00 for H_2_O and δ5.45–6.00 for urea. Subsequently, each integral region was normalized to the total sum of all integral regions for each spectrum prior to data analysis.

Multivariate data analysis was performed on the normalized NMR datasets with the software package SIMCA-P+ (version 11.0, Umetrics, Sweden). Principal component analysis (PCA) was conducted on the dataset to overview the intrinsic similarity/dissimilarity within the dataset. Projection to latent structure–discriminant analysis (PLS–DA) and orthogonal projection to latent structure–discriminant analysis (OPLS–DA) were further conducted to obtain the metabolites with significant contributions to intergroup differentiation by using unit-variance scaled NMR data as X-matrix and class information as Y-matrix [23]. The quality of the model was assessed by the parameters R^2^X and Q^2^, which represent the total explained variations for the X matrix and the model predictability, respectively. The models were certified by using a seven-fold cross validation method and a permutation test [24], [25]. A model was determined to be significant if the Q^2^ value was significant (*P* <0.05) through permutation. The metabolites associated with the group separations were indicated by coefficient-coded loading plots calculated by back transformation of the loadings. Coefficients were color coded by using MATLAB (The Mathworks Inc; Natwick, U.S.A. version 7.1) [24]. The metabolites that contributed most to the prediction of the response (class) are shown in red, whereas those that had slight/no association with the response are shown in blue. In this study, appropriate correlation coefficients were chosen as the cutoff values (depending on the number of animals used for each group) for the statistical significance based on discrimination significance (*P* <0.05). These coefficients were determined by using Pearson's product-moment correlation coefficient [24].

### Statistical analysis

The mean daily food intake, daily body weight gain, food intake/body weight gain ratio, and conventional plasma biochemical parameters were analyzed statistically by single factorial variance analysis using the general linear model procedure of SPSS 17.0 software (SPSS Inc., Chicago, IL). Datasets were performed by using post-hoc tests (LSD) for multiple comparisons to determine the statistical differences between groups, which were denoted by different letter superscripts. Data were presented as mean ± SD. The level of significance used was *P* <0.05.

## Results

### Daily food intake and bodyweight gain

The mean daily bodyweight gain of the PF group was higher than that of the WF and control groups (34.4% and 28.3%, respectively). The food intake to bodyweight gain ratio was lower in the PF group than in the WF group (*P* <0.05, Table 3).

**Table 3:** Effects of pea fiber and wheat bran fiber supplementation on body weight gain and food intake of rats.

Treatment	Control	Pea fiber	Wheat bran fiber
mean daily body weight gain (g)	2.19±0.76^a^	2.81±0.63^b^	2.09±0.84^a^
mean daily food intake (g)	14.06±1.80	14.26±1.81	13.74±2.05
food intake/body weight gain ratio	6.30±2.53^ab^	5.18±0.62^a^	7.17±3.10^b^

Data represent the means ± SD. Different superscripts indicate significant difference (*P* <0.05).

### Conventional biochemical assays

Clinical chemistry results (Table 4) demonstrated that in comparison with the control group, the PF group significantly decreased TG and TP levels (*P* <0.05). The glucose level increased in the WF group compared with the control (*P* <0.05). However, decreased AST, CRE, TG, and TP levels (*P* <0.05) and a decreasing trend in ALB level were observed in the WF group. In comparison with the WF group, the PF group showed significantly decreased ALT, AST, CRE, and TP levels, and increased ALB levels (*P* <0.05), as well as an increasing BUN trend and decreasing glucose level.

**Table 4:** Data for plasma chemistry of rats administered with control, pea fiber, and wheat bran fiber.

Parameters*	control	pea fiber	wheat bran fiber
ALB (g/L)	33.39±1.09^ab^	33.82±2.92^a^	31.51±1.69^b^
TP (g/L)	75.24±2.10^a^	71.85±1.98^b^	69.32±3.23^c^
ALB/TP	0.46±0.02	0.47±0.03	0.46±0.02
ALT	62.40±12.96^ab^	71.25±8.19^a^	54.78±9.04^b^
AST	269.90±41.47^a^	291.78±62.39^a^	219.22±33.39^b^
AST/ALT	4.38±0.39	4.31±0.72	4.02±0.28
BUN	4.64±1.07	5.26±1.14	4.20±1.24
CRE	77.89±2.89^a^	79.25±2.19^a^	69.20±5.61^b^
BUN/CRE	0.06±0.01	0.07±0.02	0.06±0.02
total cholesterol (mmol/L)	2.11±0.46	2.22±0.43	2.14±0.46
glucose (mmol/L)	3.86±1.36^a^	5.02±2.77^ab^	6.60±1.28^b^
triglycerides (mmol/L)	1.14±0.31^a^	0.88±0.20^b^	0.81±0.21^b^

* ALB, albumin; TP, total protein; ALT, alanine transaminase; AST, aspartate transaminase; BUN, blood urea bitrogen; CRE, creatinine. Data represent the means ± SD.

a,b,c Different superscripts indicate significant difference (*P* <0.05).

### ^1^H NMR spectra of urine and plasma samples

**Figure 1:**
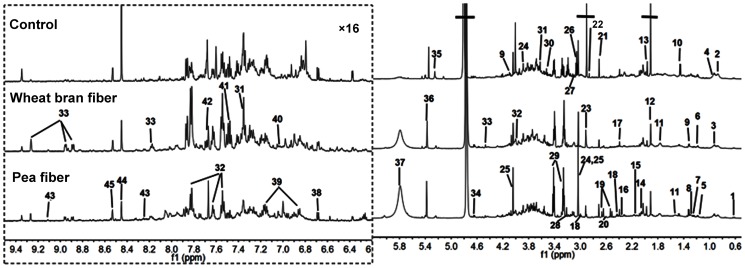
Representative one-dimensional ^1^H NMR spectra urine metabolites obtained from the (A) control, (B) pea fiber, and (C) wheat bran fiber groups. The region of δ6.2–9.5 was magnified 16 times compared with corresponding region of δ0.5–6.2 for the purpose of clarity. Metabolite keys are given in Table 1.

**Figure 2:**
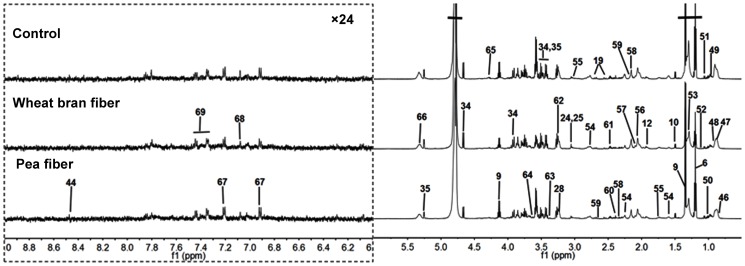
Typical 600 MHz ^1^H NMR spectra of plasma metabolites obtained from the (A) control, (B) pea fiber, and (C) wheat bran fiber groups. The region of δ6.0–9.0 was magnified 24 times compared with corresponding region of δ0.5–6.0 for the purpose of clarity. Metabolite keys are given in Table 1.

Figs. 1 and 2 respectively show the representative ^1^H NMR spectra of the urine and plasma samples taken from randomly selected rats of WF, PF, and control groups. NMR signals were assigned to specific metabolites for ^1^H resonances (Table 5). A total of 44 metabolites were unambiguously assigned for urine. The spectra of urine samples contained resonances from several amino acids, glucose, organic acids, allantoin, and choline. Tricarboxylic acid cycle metabolites, such as succinate and citrate, were also detected in the urine samples. Moreover, the plasma sample mainly contained glucose, lactate, lipids, and a series of amino acids.

**Table 5:** ^1^H NMR data for metabolites in rat urine and plasma.

keys	Metabolites	moieties	δ ^1^H (ppm) and multiplicity	samples^a^
1	bile acids	CH_3_	0.62(m), 0.75(m)	U
2	Butyrate	CH_3_	0.9(t)	U
3	α-hydroxybutyrate	CH_3_	0.94(t)	U
4	α-hydroxy-iso-valerate	δCH_3_	0.97(d)	U
5	isobutyrate	CH_3_	1.14(d)	U, P
6	Ethanol	CH_3_, CH_2_	1.19(t), 3.66(q)	U, P
7	methylmalonate	CH_3_, CH	1.26(d), 3.76(m)	U
8	α-hydroxy-n-valerate	CH_3_, γCH_2_	0.89(t), 1.31(m)	U
9	lactate	αCH, βCH_3_	4.13(q), 1.33(d)	U, P
10	alanine	αCH, βCH_3_	3.77(q), 1.48(d)	U, P
11	citrulline	γCH_2_, βCH_2_	1.56(m), 1.82(m)	U
12	acetate	CH_3_	1.92(s)	U, P
13	acetamide	CH_3_	1.99(s)	U
14	*N*-acetylglutamate	βCH_2_, γCH_2_, CH_3_	2.07(m), 1.88(m), 2.04(s)	U
15	acetone	CH_3_	2.25(s)	U, P
16	acetoacetate	CH_3_	2.3(s)	U
17	succinate	CH_2_	2.41(s)	U
18	α-ketoglutarate	βCH_2_, γCH_2_	2.45(t), 3.01(t)	U
19	citrate	CH_2_	2.55(d), 2.68(d)	U, P
20	methylamine	CH_3_	2.62(s)	U
21	dimethylamine	CH_3_	2.73(s)	U
22	trimethylamine	CH_3_	2.88(s)	U
23	dimethylglycine	CH_3_	2.93(s)	U
24	creatine	CH_3_, CH_2_	3.04(s), 3.93(s)	U, P
25	creatinine	CH_3_, CH_2_	3.04(s), 4.05(s)	U, P
26	ethanolamine	CH_2_	3.13(t)	U
27	malonate	CH_2_	3.16(s)	U
28	choline	OCH_2_, NCH_2_, N(CH_3_)_3_	4.07(t), 3.53(t), 3.20(s)	U, P
29	taurine	-CH_2_-S, -CH_2_-NH_2_	3.26(t), 3.43(t)	U
30	glycine	CH_2_	3.57(s)	U
31	phenylacetyglycine	2,6-CH, 3,5-CH, 7-CH, 10-CH	7.31(t), 7.37(m), 7.42(m), 3.68(s)	U
32	hippurate	CH_2_, 3,5-CH, 4-CH, 2,6-CH	3.97(d), 7.57(t), 7.65(t), 7.84(d)	U
33	*N*-methylnicotinamide	CH_3_, 5-CH, 4-CH, 6-CH, CH_2_	4.44(s), 8.18(d), 8.89(d), 8.96(d), 9.26(s)	U
34	β-glucose	1-CH, 2-CH, 3-CH, 4-CH, 5-CH, 6-CH	4.65(d), 3.25(dd), 3.49(t), 3.41(dd), 3.46(m), 3.73(dd), 3.90(dd)	U, P
35	α-glucose	1-CH, 2-CH, 3-CH, 4-CH, 5-CH, 6-CH	5.24(d), 3.54(dd), 3.71(dd), 3.42(dd), 3.84(m), 3.78(m)	U, P
36	allantoin	CH	5.40(s)	U, P
37	urea	NH_2_	5.82(s)	U
38	homogentisate	6-CH, 5-CH	6.7(d), 6.76(d),	U
39	*p*-hydroxyphenylacetate	6-CH, 2-CH, 3,5-CH	3.6(s), 6.87(d), 7.15(d)	U
40	*m*-hydroxyphenylacetate	6-CH, 4-CH, 3-CH	6.92(m), 7.04(d), 7.26(t)	U
41	nicotinate	2,6-CH, 4-CH, 5-CH	8.62(d), 8.25(d), 7.5(dd)	U
42	4-aminohippurate	CH_2_	7.71(d)	U
43	trigonelline	2-CH, 4-CH, 6-CH, 5-CH, CH3	9.12(s), 8.85(m), 8.83(dd), 8.19(m), 4.44(s)	U
44	formate	CH	8.46(s)	U
45	unknown		8.54(s)	U
46	HDL*	CH_3_(CH_2_)_n_	0.84(m)	P
47	LDL*	CH_3_(CH_2_)_n_	0.87(m)	P
48	VLDL*	CH_3_CH_2_CH_2_C =	0.89(t)	P
49	isoleucine	αCH, βCH, βCH_3_, γCH_2_, δCH_3_	3.68(d), 1.99(m), 1.01(d), 1.26(m), 1.47(m), 0.94(t)	P
50	leucine	αCH, βCH_2_, γCH, δCH_3_	3.73(t), 1.72(m), 1.72(m), 0.96(d), 0.97(d)	P
51	valine	αCH, βCH, γCH_3_	3.62(d), 2.28(m), 0.99(d), 1.04(d)	P
52	propionate	CH_3_, CH_2_	1.08(t), 2.18(q)	P
53	3-hydroxybutyrate	αCH_2_, βCH, γCH_3_	2.28(dd), 2.42(dd), 4.16(m), 1.20(d)	P
54	lipids (triglycerids and fatty acids)	(CH_2_)_n_, CH_2_CH_2_CO, CH_2_C = C, CH_2_CO,C = CCH_2_C = C	1.28(m),1.58(m), 2.01(m), 2.24(m), 2.76(m)	P
55	lysine	αCH, βCH_2_, γCH_2_, εCH_2_	3.76(t), 1.91(m), 1.48(m), 1.72(m), 3.01(t)	P
56	*N*-acetyl glycoprotein	CH_3_	2.04(s)	P
57	*O*-acetyl glycoprotein	CH_3_	2.08(s)	P
58	glutamate	αCH, βCH_2_, γCH_2_	3.75(m), 2.12(m), 2.35(m)	P
59	methionine	αCH, βCH_2_, γCH_2_, S-CH_3_	3.87(t), 2.16(m), 2.65(t), 2.14(s)	P
60	pyruvate	CH_3_	2.37(s)	P
61	glutamine	αCH, βCH_2_, γCH_2_	3.78(m), 2.14(m), 2.45(m)	P
62	glycerolphosphocholine	CH_3_, βCH_2_, αCH_2_	3.22(s), 3.69(t), 4.33(t)	P
63	phosphorylcholine	N(CH_3_)_3_, OCH_2_, NCH_2_	3.22(s), 4.21(t), 3.61(t)	P
64	*myo*-inositol	1,3-CH, 2-CH, 5-CH, 4,6-CH	3.60(dd), 4.06(t), 3.30(t), 3.63(t)	P
65	threonine	αCH, βCH, γCH_3_	3.58(d), 4.24(m), 1.32(d)	P
66	unsaturated lipids	= CH-CH_2_C = , -CH = CH-	5.19 (m), 5.30(m)	P
67	tyrosine	2,6-CH, 3,5-CH	7.20(dd), 6.91(d)	P
68	1-methylhistidine	4-CH, 2-CH	7.05(s), 7.78(s)	P
69	phenylalanine	2,6-CH, 3,5-CH, 4-CH	7.32(m), 7.42(m), 7.37(m)	P
70	3-methylhistidine	4-CH, 2-CH	7.07(s), 7.67(s)	P

a U, urine; P, plasma; * HDL, high density lipoprotein; LDL, low density lipoprotein; VLDL, low density lipoprotein; s, singlet; d, doublet; t, triplet; q, quartet; dd, doublet of doublets; m, multiplet.

### Multivariate data analysis of NMR data

**Figure 3:**
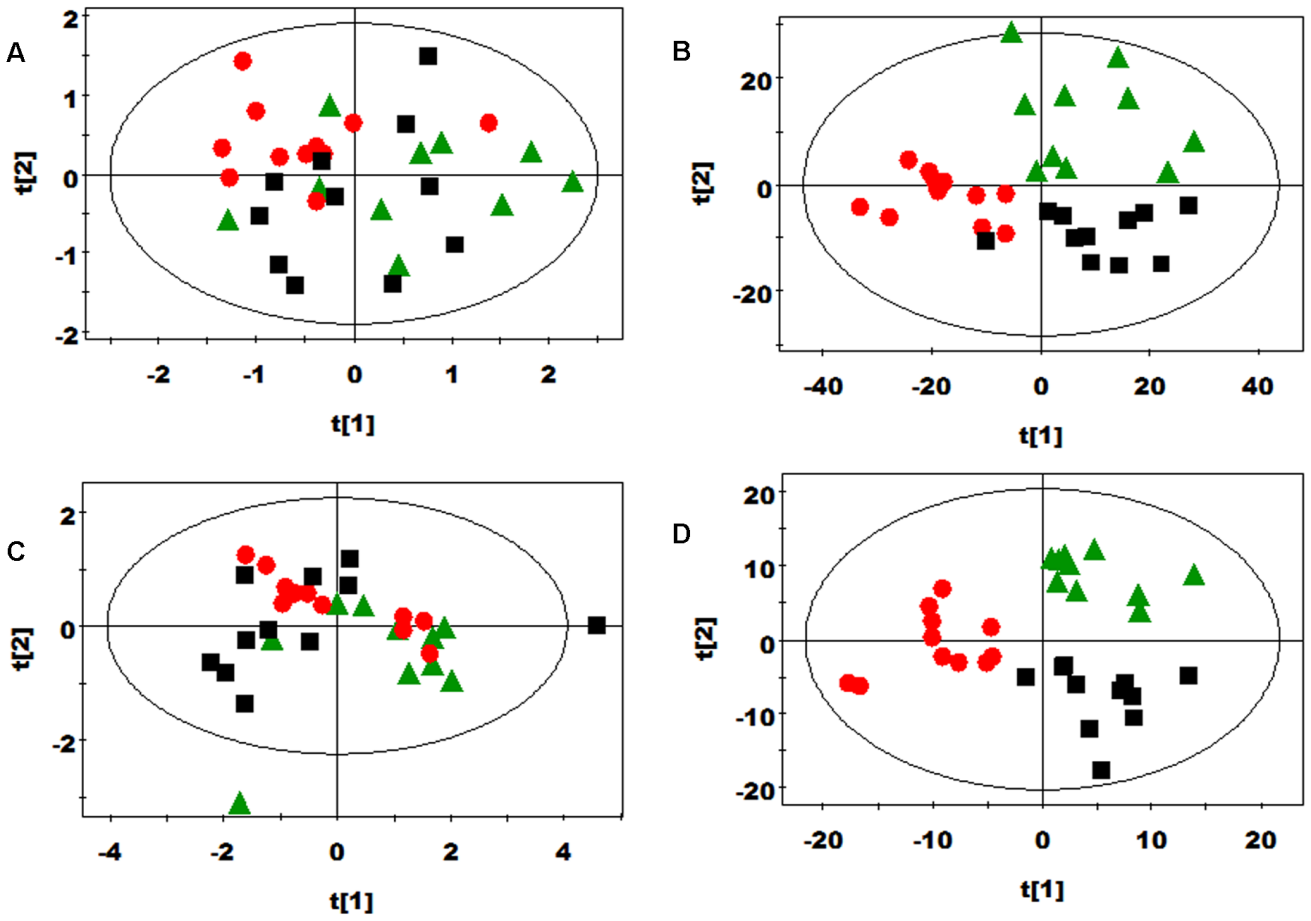
(A) PCA (R^2^X = 0.917, Q^2^ = 0.815) and (B) PLS-DA (R^2^X = 0.173, Q^2^ = 0.103) score plots based on the ^1^H NMR spectra of plasma metabolites obtained from the control (black squares), pea fiber (green triangles), and wheat bran fiber groups (red circles). One plasma sample from pea fiber group was excluded because it positioned outside the Hotelling's T^2^ elipse on the score plot. (C) PCA (R^2^X = 0. 477, Q^2^ = 0.242) and (D) PLS-DA score plots (R^2^X = 0.724, R^2^Y = 0.983, Q^2^ = 0.673) based on the ^1^H NMR spectra of the urine obtained from urinary metabolites obtained from the control (black squares), pea fiber (green triangles), and wheat bran fiber groups (red circles). One urinary sample from pea fiber group was excluded because it positioned outside the Hotelling's T^2^ elipse on the PCA and PLS-DA score plot.

**Figure 4:**
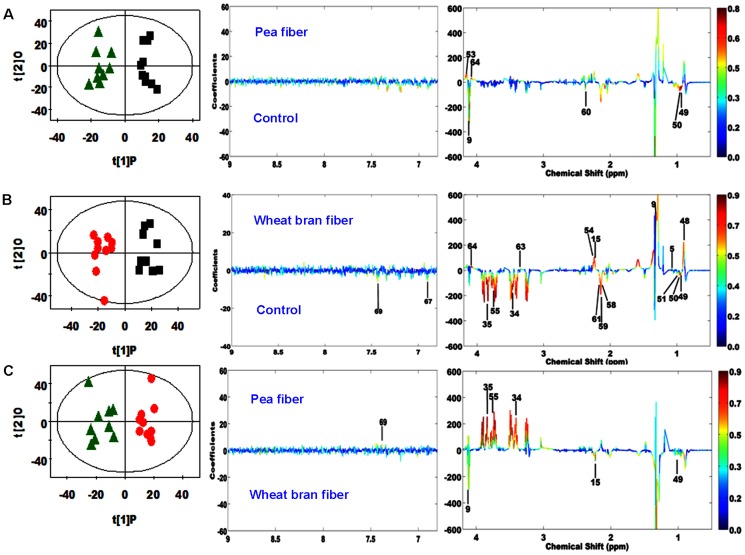
OPLS–DA scores plots (left panel) and the corresponding coefficient loading plots (right panel) of plasma metabolites derived from the control (black squares), pea fiber (green triangles), and wheat bran fiber groups (red circles). (A: R^2^X = 20.5%, Q^2^ = 0.371; B: R^2^X = 28.6%, Q^2^ = 0.626; C: R^2^X  = 28.3%, Q^2^  = 0.289). Two plasma samples from pea fiber group, one sample from wheat bran fiber, and one sample from control were excluded because they positioned outside the Hotelling's T^2^ elipse on the score plot. The color map shows the significance of metabolite variations between the two classes. The peaks in the positive direction indicate the metabolites that are more abundant in the groups in the positive direction of the first principal component. The metabolites that are more abundant in the groups in the negative direction of the first primary component are presented as peaks in the negative direction.

**Figure 5:**
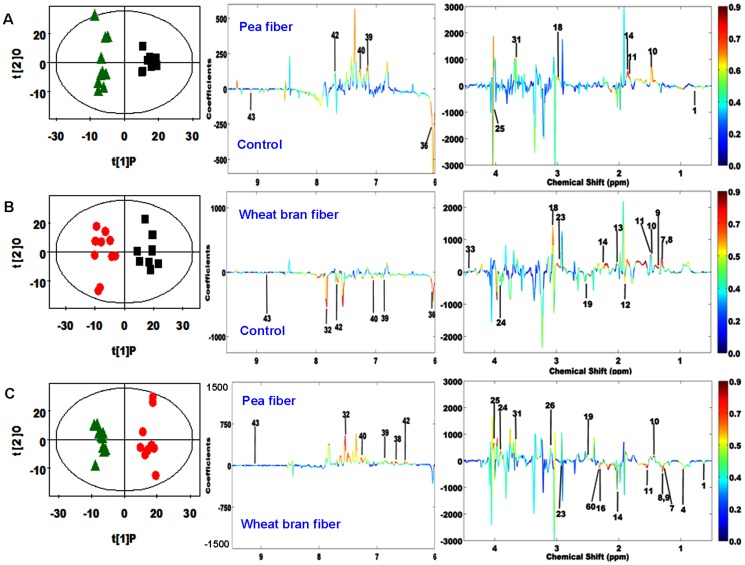
OPLS–DA scores plots (left panel) and the corresponding coefficient loading plots (right panel) of urinary metabolites derived from the control (black squares), pea fiber (green triangles), and wheat bran fiber groups (red circles). (A: R^2^X = 29.3%, Q^2^ = 0.76; B: R^2^X = 38.6%, Q^2^ = 0.819; C: R^2^X  = 33.7%, Q^2^  = 0.794). One urinary sample from pea fiber group, one sample from wheat bran fiber, and two samples from control group were excluded because they positioned outside the Hotelling's T^2^ elipse on the score plot. The color map shows the significance of metabolite variations between the two classes. The peaks in the positive direction indicate the metabolites that are more abundant in the groups in the positive direction of the first principal component. The metabolites that are more abundant in the groups in the negative direction of the first primary component are presented as peaks in the negative direction.

PCA was initially performed on the plasma spectral data. Two principal components were calculated for the treatment groups, with 46.0% and 26.6% of the variables explained by PC1 and PC2, respectively. PCA results (Fig. 3A) showed that separations in rats from the WF, PF, and control groups were absent in their metabolic plasma profiles. Furthermore, the plasma metabolic changes in the rats from the three groups were analyzed by using OPLS–DA. The corresponding coefficient analysis showed that PF significantly increased the plasma levels of 3-hydroxybutyrate and *myo*-inositol and decreased the plasma levels of isoleucine, leucine, lactate, and pyruvate compared with the control group (*P* <0.05, Fig. 4A and Table 6). Moreover, WF significantly increased the plasma levels of acetone, isobutyrate, lactate, *myo*-inositol, phosphorylcholine, VLDL, lipid (triglycerides and fatty acids), and unsaturated lipid and decreased the plasma levels of glutamine, glutamate, isoleucine, leucine, lysine, methionine, phenylalanine, tyrosine, valine, α-glucose, and β-glucose compared with the control group (*P* <0.05, Fig. 4B and Table 6). In comparison with the WF group, the PF group showed significantly increased plasma levels of lysine, phenylalanine, α-glucose, and β-glucose and decreased plasma levels of acetone, allantoin, isoleucine, and lactate (*P* <0.05, Fig. 4C and Table 6). PLS–DA was conducted on the urine spectra of the WF, PF, and control groups. The score plots (Fig. 3B) highlighted three clusters corresponding to the three groups. The metabolic profiles of WF, PF, and control group were compared by using OPLS–DA to further identify the important urine metabolic changes induced by fiber supplementation. Multivariate data analysis showed that the urine levels of 4-aminohippurate, alanine, citrulline, *m*-hydroxyphenylacetate, *N*-acetylglutamate, phenylacetyglycine, *p*-hydroxyphenylacetate, and α-ketoglutarate were higher in the PF group than in the control group (*P* <0.05). By contrast, the urine levels of allantoin, bile acids, creatinine, and trigonelline were lower in the PF group than in the control group (*P* <0.05, Fig. 5A and Table 7). The metabolic profile of the WF group was compared with that of the control group by using OPLS–DA to observe the effect of WF supplementation. The urine levels of acetamide, alanine, citrulline, lactate, methylmalonate, dimethylglycine (DMG), *N*-acetylglutamate, *N*-methylniconamide, and α-ketoglutarate were significantly higher in the WF group than in the control group (*P* <0.05). By contrast, the urine levels of 4-aminohippurate, acetate, allantoin, citrate, creatine, hippurate, *m*-hydroxyphenylacetate, *p*-hydroxyphenylacetate, and trigonelline were lower in the WF group than in the control group (*P* <0.05, Fig. 5B and Table 7). OPLS–DA was also carried out to determine the degree of influence of PF supplementation on metabolism compared with the WF group. The urine levels of 4-aminohippurate, alanine, citrate, creatine, creatinine, ethanolomine, hippurate, homogentisate, *m*-hydroxyphenylacetate, phenylacetyglycine, *p*-hydroxyphenylacetate, and trigonelline were significantly higher in the PF group than in the WF group (*P* <0.05). By contrast, the urine levels of acetoacetate, bile acids, citrulline, lactate, methylmalonate, DMG, and *N*-acetylglutamate were lower in the PF group than in the WF group (*P* <0.05, Fig. 5C and Table 7).

**Table 6:** OPLS–DA coefficients derived from the NMR data of plasma metabolites obtained from the (A) control, (B) pea fiber, and (C) wheat bran fiber groups.

metabolite	B (vs A)^a^	C (vs A)^a^	B (vs C)^a^
isoleucine (49)	−0.789	−0.792	−0.692
leucine (50)	−0.757	−0.694	–
3-hydroxybutyrate (53)	0.652	–	–
lactate (9)	−0.658	0.732	−0.677
*myo*-inositol (64)	0.671	0.823	–
pyruvate (60)	−0.686	–	–
acetone (15)	–	0.784	−0.844
glutamate (58)	–	−0.652	–
glutamine (61)	–	−0.705	–
allantoin (36)	–	–	−0.676
isobutyrate (5)	–	0.611	–
VLDL (48)	–	0.669	–
lipid (54)	–	0.737	–
unsaturated lipids (66)	–	0.666	–
lysine (55)	–	−0.780	0.694
methionine (59)	–	−0.767	–
phosphorylcholine (63)	–	0.660	–
phenylalanine (69)	–	−0.615	0.697
tyrosine (67)	–	−0.606	–
valine (51)	–	−0.605	–
α-glucose (35)	–	−0.876	0.847
β-glucose (34)	–	−0.874	0.857

a Correlation coefficients: positive and negative signs indicate positive and negative correlation in the concentrations, respectively. The correlation coefficient of |r| > 0.632 (for B vs A and B vs C) or 0.602 (for C vs A) was used as the cutoff value. “–” means the correlation coefficient |r| is less than 0.632 (for B vs A and B vs C) or 0.602 (for C vs A).

**Table 7:** OPLS–DA coefficients derived from the NMR data of urine metabolites obtained from the (A) control, (B) pea fiber, and (C) wheat bran fiber groups.

metabolite	B (vs A)^a^	C (vs A)^a^	B (vs C)^a^
4-aminohippurate (42)	0.668	−0.724	0.824
alanine (10)	0.797	0.707	0.814
allantoin (36)	−0.722	−0.887	–
bile acids (1)	−0.750	–	−0.671
citrulline (11)	0.785	0.911	−0.878
creatinine (25)	−0.735	–	0.807
*m*-hydroxyphenylacetate (40)	0.734	−0.707	0.753
*N*-acetylglutamate (14)	0.799	0.853	−0.722
phenylacetyglycine (31)	0.794	–	0.764
*p*-hydroxyphenylacetate (39)	0.874	−0.679	0.718
trigonelline (43)	–0.832	−0.872	0.719
α-ketoglutarate (18)	0.712	0.792	–
acetamide (13)	–	0.709	–
acetate (12)	–	−0.675	–
citrate (19)	–	−0.743	0.646
creatine (24)	–	−0.761	0.706
hippurate (32)	–	−0.877	0.809
lactate (9)	–	0.813	−0.712
methylmalonate (7)	–	0.702	−0.693
dimethylglycine (23)	–	0.748	–0.643
*N*-methylnicotinamide (33)	–	0.690	–
α-hydroxy-n-valerate (8)	–	0.864	−0.842
acetoacetate (16)	–	–	−0.687
ethanolamine (26)	–	–	0.636
homogentisate (38)	–	–	0.810
α-hydroxy-iso-valerate (4)	–	–	−0.636

a Correlation coefficients: positive and negative signs indicate positive and negative correlation in the concentrations, respectively. The correlation coefficient of |r| > 0.666 (for B vs A and C vs A) or 0.632 (for B vs C) was used as the cutoff value. “−” means the correlation coefficient |r| is less than 0.666 (for B vs A and C vs A) or 0.632 (for B vs C).

## Discussion

### Antioxidant activity

Both PF and WF has antioxidant activity. In this study, urinary allantoin levels were decreased by PF and WF. Allantoin is a product of purine metabolism in most mammals. Allantoin in urine can be generated through non-enzymatic means via high levels of reactive oxygen species. Thus, allantoin can be used as a marker of oxidative stress [26], [27]. Moreover, the levels of urinary *m*-hydroxyphenylacetate, which has a protective biological activity in animals, were increased by PF. The loss of water molecule from creatine leads to the formation of creatinine. Creatinine is transported to the kidneys by blood plasma and eliminated from the body by glomerular filtration and partial tubular excretion. Creatinine is generally produced at a fairly constant rate by the body [27]. In this study, the PF group has lower urinary creatinine levels compared with the control group. Oxidative stress increases the urinary excretion of creatinine [28], and studies on the effects of antioxidant in rabbits and human suggest a decrease in urinary creatinine levels [29]. This decrease in urinary creatinine level following PF administration is possibly caused by the antioxidative activity of PF. Thus, PF can enhance the antioxidant status in rats. Urinary DMG levels were increased by WF. DMG is produced in the cells as an intermediate in choline to glycine metabolism. DMG also acts as a detoxifying agent and antioxidant, protecting body cells from unwanted reactions induced by free radicals. DMG was also claimed to be an energy booster and a stress reducer [27], [30]. Thus, the elevation of urinary DMG level indicates that reactive oxygen species production may be decreased in rats. Furthermore, an elevated level of urinary *N-*methylnicotinamide was observed in the WF group. *N-*methylnicotinamide is the methylated metabolite of nicotinamide, which can be produced during the conversion of *S*-adenosylmethionine to *S*-adenosylhomocysteine in the biosynthesis of cysteine, an essential amino acid of glutathione synthesis [31]. Thus, WF can increase antioxidant status in rats. This finding is in agreement with previous study in which whole grain wheat flour (versus refined wheat flour) improves the liver redox status [16]. Therefore, PF and WF have antioxidant status in rats. This result may be attributed to the antioxidant compounds of DF such as ferulic acid, lignins, phytic acid, zinc, copper, selenium, and manganese in the grain envelope and vitamin E in the germ [32]. To our knowledge, studies on the antioxidant effects of PF are scarce in animals.

### Bile acid and lipid metabolism

PF can alter bile acid metabolism. Bile acids are formed from cholesterol in the liver and secreted through the bile into the intestine where they facilitate the formation of micelles, which enhances the processing of dietary fat. Bile acids also increase the biliary excretion of unmetabolized cholesterol into the bile [27]. In this study, urinary bile acid levels were decreased by PF. The decreased urinary bile acid is in agreement with previous study, which denotes that the total excretion of bile acids is reduced. The concentration of fecal bile acids is lower in PF compared with the fiber-free diet [4]. The fiber can also decrease absorption of dietary cholesterol [4]. The possible inhibition of cholesterol synthesis by SCFAs caused by colonic fermentation has been proposed as a mechanism for the cholesterol-lowering effect of fiber [4]. The decreased absorption of bile acids caused by their binding to DF in the intestinal lumen is a possible mechanism by which DF decreases blood lipid levels [4]. The decrease in urinary bile acids indicates that the absorption of dietary fat is decreased. This result is in agreement with the previous study that PF significantly increases the amount of fecal fat by 9% to 56% [4]. This finding is in accordance with the result of the present study, in which PF decreased plasma triglyceride levels. WF decreased plasma triglyceride levels. However, urinary bile acid levels were not significantly affected by WF. Previous studies suggest that wheat bran decreases fecal bile acid excretion concentrations [12], [33]. The bile acids detected in plasma and urine had no significant differences in the WF group. Therefore, the result of this study indicates that bile acids in WF may not be reflected in plasma or urine concentrations.

PF supplementation can also decrease lipid oxidation. In this study, PF improved plasma 3-hydroxybutyrate levels. The levels of urinary ketone bodies such as acetone and acetoacetate also decreased in the PF group compared with the WF group. Ketone bodies are the products of β-oxidation of fatty acid in the mitochondria. A decrease in these biochemical levels suggests that PF decreases the β-oxidation of fatty acids more compared with the WF group. Acetoacetate and 3-hydroxybutyrate are products of fatty acid oxidation in the liver, and their ratios are useful indicators of the mitochondrial redox state [34]. PF supplementation decreased the urine level of acetoacetate, but had no effect on 3-hydroxybutyrate compared with the WF group. Thus, the acetoacetate/3-hydroxybutyrate ratio also decreased. This result suggests a less oxidized state of the cells. This phenomenon, which may be caused by the antioxidant components from fiber that decreases lipid peroxidation, is the result of the decreased oxidation of fatty acids. To the best of our knowledge, studies on the lipid peroxidation of PF in animals are limited. Moreover, WF can affect lipid metabolism and increase plasma acetone, VLDL, lipid (triglycerides and fatty acids), and unsaturated lipid levels in rats. Therefore, WF can change lipid metabolism. PF and WF consumption can alter the concentrations of lipid signaling molecules in rats. The plasma concentrations of *myo*-inositol were elevated in response to PF and WF supplementation. This carbocyclic polyol plays a critical role in the structural basis for a number of secondary messengers (including inositol phosphates, phosphatidylinositol, and phosphatidyl inositol phosphate) in eukaryotic cells [35]. Consequently, inositol is related with the regulation of intracellular calcium concentrations, insulin signal transduction, gene expression, and oxidation of fatty acids [35], [36]. Moreover, phosphorylcholine was increased, and phosphorylcholine/glycerolphosphocholine (data not shown) was decreased in the WF group compared with the control group. Phosphorylcholine and glycerolphosphocholine have important functions in cell metabolism and signaling processes, which is attributed to the modification of the structural integrity of the cell membrane [37], [38]. Glycerolphosphocholine and phosphorylcholine crucially function in lipid cholesterol transport and metabolism [38]. Furthermore, the gross energy and crude protein values in the test diets measured were similar in all diets. Here, the fat content in the control diet was higher than the tests diets. The difference was reflected in the higher TG values in the control diet. Thus, fiber differences need further attention in the future. Collectively, PF and WF can alter the lipid metabolism in rats.

### Glucose and energy metabolism

PF can decrease plasma glycolytic metabolite (pyruvate) and lactate levels and increase the urinary alanine levels in rats. This finding suggests that anaerobic glycolysis and glycogenolysis were decreased. However, the WF group exhibited a significant decrease of plasma glucose compared with the control or PF group. Glucose is a major substrate that provides energy for animal growth and development. This finding is consistent with that of previous study [13]. Increased lactate concentration was also observed in the urine and plasma of the WF group. Lactate is associated with energy metabolism and is the end product of compounds in relation to energy metabolism. Increased lactate level is linked with increased anaerobic glycolysis. In addition, increased plasma lactate level implies the inhibition of gluconeogenesis and the modification of carbohydrate and energy metabolism. Furthermore, WF can increase urinary alanine levels in rats, thus suggesting that glycogenolysis was decreased. These findings indicate that WF exposure can promote glycolysis and can decrease glycogenolysis. The decreased glycolysis in PF and increased glycolysis in WF may be attributed to the different fiber diet administered. Moreover, PF and WF can improve urinary α-ketoglutarate and alanine levels, respectively. The urinary citrate levels decreased in the WF group compared with the control group but increased in the PF group compared with the WF group. Considering this result, the tricarboxylic acid cycle was altered in rats. The creatine levels decreased in WF compared with the control group. However, the creatine levels increased in the PF group compared with the WF group. Creatine supplies energy to muscles in vertebrates in the form of stored creatine phosphate. The creatine levels in the animals are synthesized de novo in the liver by the use of amino acids, such as arginine, glycine, and methionine. Therefore, PF and WF consumption can affect energy metabolism in rats.

### Amino acid metabolism

The exposure in PF and WF can alter amino acid metabolism. In the present study, plasma TP and tyrosine (involve in protein synthesis) were decreased by WF supplementation, which implies that WF inhibits protein synthesis. This result is in agreement with that of previous study, which states that wheat bran can reduce nitrogen utilization in rats [39]. Consequently, more amino acids were decreased in protein synthesis, leading to decreased levels of the amino acids present in plasma. In this research, the levels of plasma lysine, methionine, glutamate, and glutamine were reduced, which agrees with the function of WF in decreasing the protein synthesis in rats. Glutamine activates signaling pathways to promote protein synthesis and eventually animal growth and development. Moreover, results showed that levels of branched-chain amino acids were decreased by PF and WF supplementation. These amino acids are recognized as key metabolites associated with protein synthesis and cell growth. Furthermore, urinary citrulline and *N*-acetylglutamate levels were increased by PF and WF consumption. Citrulline is an amino acid produced from ornithine and carbamoyl phosphate in one of the central reactions in the urea cycle. This amino acid is obtained from arginine as a by-product of the reaction catalyzed by the NOS family. In this reaction, arginine is first oxidized into *N*-hydroxyl-arginine and oxidized further to citrulline in conjunction with the release of nitric oxide [27]. Urea has a critical function in the metabolism of nitrogen-containing compounds. *N*-acetylglutamate is required for the normal function of the urea cycle, and the variations in its concentration affect urea production rate and other substrates for urea synthesis [40]. In comparison with the WF group, the PF group showed an increasing trend in BUN. This finding is in accordance with the present study, which denotes that PF leads to more daily body weight. Moreover, PF decreased urinary *N*-acetylglutamate levels compared with WF. An increase in BUN was accompanied with a decrease in *N*-acetylglutamate, which indicates that urea production is regulated by *N*-acetylglutamate. Moreover, PF decreased the plasma TP levels, and WF decreased the plasma ALB levels. These findings suggest that PF and WF consumption can affect amino acid metabolism in rats.

### Gut microbiota metabolism

The exposure in PF and WF can modify gut microbiota metabolism. The energy providers for the metabolism in the colon are SCFAs (e.g., isobutyrates and acetate), which are produced by bacteria in the colon through fermentation of unabsorbed DF. In this study, isobutyrates were increased, and acetate was decreased in the WF group. This finding is caused by the possibility that gut microbiota can either manufacture or utilize these products. The results of this study also indicate that WF decreased the urinary excretion of hippurate, which is produced through both renal and hepatic syntheses of glycine and benzoic acid. Hippurate is the degradation product of flavonols acted upon by intestinal microorganisms [41]. Consequently, a change in the excretion of this compound suggests a shift in the functional metabolism of the microbiota. Variations in urinary hippurate concentration have also been correlated with the changes in the distribution of intestinal microbial colonies [42]. The modified levels of gut microbial cometabolites such as phenylacetylglycine and *p*-hydroxyphenylacetate in PF exposure and gut microbial cometabolites including *p*-hydroxyphenylacetate in WF confirmed the association of the disturbance to gut microbiota. Phenylacetate was transformed from phenylalanine via the action of gut microbiota; phenylacetate was then conjugated with glycine to form phenylacetylglycine [42]. Previous reports suggest that elevated levels of urinary phenylacetylglycine are shown in the abnormal accumulation of phospholipids in the liver of rats, and these levels act as a surrogate biomarker for associated changes in the gut microbiota [43]. Acyl-CoA has important function in glycine conjugation [44]; however, whether this enzyme is regulated by PF exposure remains uncertain. *p*-Hydroxyphenylacetate is a metabolite of tyrosine through enteric bacteria. Mammalian metabolism is significantly affected by its interaction with the complex gut microbiota [45]. Urinary hippurate and *p*-hydroxyphenylacetate levels were decreased by WF, however, urinary phenylacetylglycine and *p*-hydroxyphenylacetate were increased by PF. The possible reason for this finding is the fiber dietary differences. The introduction of PF and WF supplementation into the mammalian system may displace baseline mammalian-to-microbial behavior, thus causing a disruption in microbial populations and eventually in metabolism. The changes in these metabolites are attributed to reduced number and/or altered activity of intestinal microorganisms. PF and WF have been shown to selectively regulate intestinal bacterial activity including stimulating the growth of health-promoting bacterial species (*Bifidobacteria* and *Lactobacilli*) and suppressing the growth of potential pathogenic bacteria species (*Escherichia Coli*) [46]. Gut microbiota can significantly affect the development and structure of the intestinal epithelium, the digestive and absorptive capabilities of the intestine, and the host immune system [47]. Thus, the disturbances of gut microbiota caused by the supplementation of PF and WF can improve gut health status.

In conclusion, PF and WF exposure affects the urine and plasma metabolome of rats. The effects of PF and WF in the metabolic profiles are more pronounced in the urine than in the plasma, where many fiber diet-derived metabolites were measured. The consumption of PF and WF can promote antioxidant activity and change some common systemic metabolic processes, including lipid metabolism, glycogenolysis and glycolysis metabolism, energy metabolism, protein biosynthesis, and gut microbiota metabolism. PF can also decrease bile acid metabolism. The metabolic profiles of the rats exposed to PF and WF can improve the present understanding of the metabolic status of PF and WF. This research also contributes in defining the effects of metabolic modifiers and in refining nutritional requirements to provide better nutritional support for growth and health. This study emphasizes the potential metabolomic strategy in the assessment of nutritional interventions in a mammalian system. To the best of our knowledge, this is the first study to systematically identify the expressed metabolites in urine and plasma from PF and WF supplementation. Future studies may be directed toward a mechanistic understanding on the effects of PF and WF in animal tissue intermediary metabolism.
