# Correction: Pea Fiber and Wheat Bran Fiber Show Distinct Metabolic Profiles in Rats as Investigated by a ^1^H NMR-Based Metabolomic Approach

**DOI:** 10.1371/journal.pone.0119117

**Published:** 2015-03-05

**Authors:** The PLOS ONE Staff

There are errors in the Author Contributions. The correct contributions are: Conceived and designed the experiments: GL LX TF YC GJ HZ JW XC CW. Performed the experiments: GL LX TF JW. Analyzed the data: GL LX TF. Contributed reagents/materials/analysis tools: GL. Wrote the paper: GL.

Additionally, there is an error in the legends for Fig. 1 and Fig. 2. “Table 1” should say “Table 5.” Please view the complete, correct Fig. 1 and Fig. 2 legends below.

**Fig 1:**
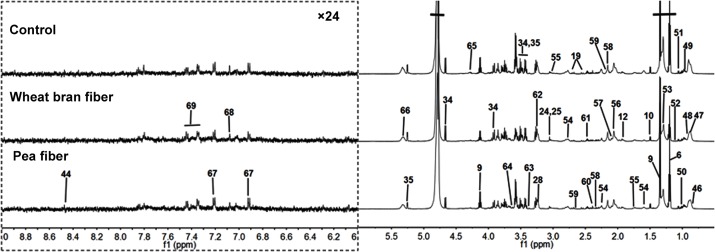
Representative one-dimensional ^1^H NMR spectra urine metabolites obtained from the (A) control, (B) pea fiber, and (C) wheat bran fiber groups. The region of δ6.2–9.5 was magnified 16 times compared with corresponding region of δ0.5–6.2 for the purpose of clarity. Metabolite keys are given in Table 5.

**Fig 2:**
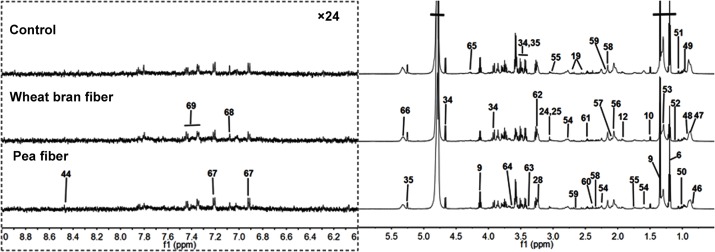
Typical 600 MHz ^1^H NMR spectra of plasma metabolites obtained from the (A) control, (B) pea fiber, and (C) wheat bran fiber groups. The region of δ6.0–9.0 was magnified 24 times compared with corresponding region of δ0.5–6.0 for the purpose of clarity. Metabolite keys are given in Table 5.

Further, there is an error in the footnotes for Table 5. VLDL stands for “very low density lipoprotein,” not “low density lipoprotein.” Please view the complete, correct Table 5 footnotes below.

**Table 5:** ^1^H NMR data for metabolites in rat urine and plasma.

keys	Metabolites	moieties	δ ^1^H (ppm) and multiplicity	samples^a^
1	bile acids	CH_3_	0.62(m), 0.75(m)	U
2	Butyrate	CH_3_	0.9(t)	U
3	α-hydroxybutyrate	CH_3_	0.94(t)	U
4	α-hydroxy-iso-valerate	δCH_3_	0.97(d)	U
5	isobutyrate	CH_3_	1.14(d)	U, P
6	Ethanol	CH_3_, CH_2_	1.19(t), 3.66(q)	U, P
7	methylmalonate	CH_3_, CH	1.26(d), 3.76(m)	U
8	α-hydroxy-n-valerate	CH_3_, γCH_2_	0.89(t), 1.31(m)	U
9	lactate	αCH, βCH_3_	4.13(q), 1.33(d)	U, P
10	alanine	αCH, βCH_3_	3.77(q), 1.48(d)	U, P
11	citrulline	γCH_2_, βCH_2_	1.56(m), 1.82(m)	U
12	acetate	CH_3_	1.92(s)	U, P
13	acetamide	CH_3_	1.99(s)	U
14	*N*-acetylglutamate	βCH_2_, γCH_2_, CH_3_	2.07(m), 1.88(m), 2.04(s)	U
15	acetone	CH_3_	2.25(s)	U, P
16	acetoacetate	CH_3_	2.3(s)	U
17	succinate	CH_2_	2.41(s)	U
18	α-ketoglutarate	βCH_2_, γCH_2_	2.45(t), 3.01(t)	U
19	citrate	CH_2_	2.55(d), 2.68(d)	U, P
20	methylamine	CH_3_	2.62(s)	U
21	dimethylamine	CH_3_	2.73(s)	U
22	trimethylamine	CH_3_	2.88(s)	U
23	dimethylglycine	CH_3_	2.93(s)	U
24	creatine	CH_3_, CH_2_	3.04(s), 3.93(s)	U, P
25	creatinine	CH_3_, CH_2_	3.04(s), 4.05(s)	U, P
26	ethanolamine	CH_2_	3.13(t)	U
27	malonate	CH_2_	3.16(s)	U
28	choline	OCH_2_, NCH_2_, N(CH_3_)_3_	4.07(t), 3.53(t), 3.20(s)	U, P
29	taurine	-CH_2_-S, -CH_2_-NH_2_	3.26(t), 3.43(t)	U
30	glycine	CH_2_	3.57(s)	U
31	phenylacetyglycine	2,6-CH, 3,5-CH, 7-CH, 10-CH	7.31(t), 7.37(m), 7.42(m), 3.68(s)	U
32	hippurate	CH_2_, 3,5-CH, 4-CH, 2,6-CH	3.97(d), 7.57(t), 7.65(t), 7.84(d)	U
33	*N*-methylnicotinamide	CH_3_, 5-CH, 4-CH, 6-CH, CH_2_	4.44(s), 8.18(d), 8.89(d), 8.96(d), 9.26(s)	U
34	β-glucose	1-CH, 2-CH, 3-CH, 4-CH, 5-CH, 6-CH	4.65(d), 3.25(dd), 3.49(t), 3.41(dd), 3.46(m), 3.73(dd), 3.90(dd)	U, P
35	α-glucose	1-CH, 2-CH, 3-CH, 4-CH, 5-CH, 6-CH	5.24(d), 3.54(dd), 3.71(dd), 3.42(dd), 3.84(m), 3.78(m)	U, P
36	allantoin	CH	5.40(s)	U, P
37	urea	NH_2_	5.82(s)	U
38	homogentisate	6-CH, 5-CH	6.7(d), 6.76(d),	U
39	*p*-hydroxyphenylacetate	6-CH, 2-CH, 3,5-CH	3.6(s), 6.87(d), 7.15(d)	U
40	*m*-hydroxyphenylacetate	6-CH, 4-CH, 3-CH	6.92(m), 7.04(d), 7.26(t)	U
41	nicotinate	2,6-CH, 4-CH, 5-CH	8.62(d), 8.25(d), 7.5(dd)	U
42	4-aminohippurate	CH_2_	7.71(d)	U
43	trigonelline	2-CH, 4-CH, 6-CH, 5-CH, CH3	9.12(s), 8.85(m), 8.83(dd), 8.19(m), 4.44(s)	U
44	formate	CH	8.46(s)	U
45	unknown		8.54(s)	U
46	HDL*	CH_3_(CH_2_)_n_	0.84(m)	P
47	LDL*	CH_3_(CH_2_)_n_	0.87(m)	P
48	VLDL*	CH_3_CH_2_CH_2_C =	0.89(t)	P
49	isoleucine	αCH, βCH, βCH_3_, γCH_2_, δCH_3_	3.68(d), 1.99(m), 1.01(d), 1.26(m), 1.47(m), 0.94(t)	P
50	leucine	αCH, βCH_2_, γCH, δCH_3_	3.73(t), 1.72(m), 1.72(m), 0.96(d), 0.97(d)	P
51	valine	αCH, βCH, γCH_3_	3.62(d), 2.28(m), 0.99(d), 1.04(d)	P
52	propionate	CH_3_, CH_2_	1.08(t), 2.18(q)	P
53	3-hydroxybutyrate	αCH_2_, βCH, γCH_3_	2.28(dd), 2.42(dd), 4.16(m), 1.20(d)	P
54	lipids (triglycerids and fatty acids)	(CH_2_)_n_, CH_2_CH_2_CO, CH_2_C = C, CH_2_CO，C = CCH_2_C = C	1.28(m)，1.58(m), 2.01(m), 2.24(m), 2.76(m)	P
55	lysine	αCH, βCH_2_, γCH_2_, εCH_2_	3.76(t), 1.91(m), 1.48(m), 1.72(m), 3.01(t)	P
56	*N*-acetyl glycoprotein	CH_3_	2.04(s)	P
57	*O*-acetyl glycoprotein	CH_3_	2.08(s)	P
58	glutamate	αCH, βCH_2_, γCH_2_	3.75(m), 2.12(m), 2.35(m)	P
59	methionine	αCH, βCH_2_, γCH_2_, S-CH_3_	3.87(t), 2.16(m), 2.65(t), 2.14(s)	P
60	pyruvate	CH_3_	2.37(s)	P
61	glutamine	αCH, βCH_2_, γCH_2_	3.78(m), 2.14(m), 2.45(m)	P
62	glycerolphosphocholine	CH_3_, βCH_2_, αCH_2_	3.22(s), 3.69(t), 4.33(t)	P
63	phosphorylcholine	N(CH_3_)_3_, OCH_2_, NCH_2_	3.22(s), 4.21(t), 3.61(t)	P
64	*myo*-inositol	1,3-CH, 2-CH, 5-CH, 4,6-CH	3.60(dd), 4.06(t), 3.30(t), 3.63(t)	P
65	threonine	αCH, βCH, γCH_3_	3.58(d), 4.24(m), 1.32(d)	P
66	unsaturated lipids	= CH-CH_2_C =, -CH = CH-	5.19 (m), 5.30(m)	P
67	tyrosine	2,6-CH, 3,5-CH	7.20(dd), 6.91(d)	P
68	1-methylhistidine	4-CH, 2-CH	7.05(s), 7.78(s)	P
69	phenylalanine	2,6-CH, 3,5-CH, 4-CH	7.32(m), 7.42(m), 7.37(m)	P
70	3-methylhistidine	4-CH, 2-CH	7.07(s), 7.67(s)	P

^a^ U, urine; P, plasma;

* HDL, high density lipoprotein; LDL, low density lipoprotein; VLDL, very low density lipoprotein; s, singlet; d, doublet; t, triplet; q, quartet; dd, doublet of doublets; m, multiplet

## References

[1] Liu G, Xiao L, Fang T, Cai Y, Jia G, Zhao H, et al (2014) Pea Fiber and Wheat Bran Fiber Show Distinct Metabolic Profiles in Rats as Investigated by a ^1^H NMR-Based Metabolomic Approach. PLoS ONE 9(12): e115561 doi:10.1371/journal.pone.0115561 25541729 10.1371/journal.pone.0115561 PMC4277351

